# Executive poverty experience and innovation performance: A study of moderating effects and influencing mechanism

**DOI:** 10.3389/fpsyg.2022.946167

**Published:** 2022-08-01

**Authors:** Ximeng Jia, Tao Wang, Chen Chen

**Affiliations:** ^1^Business School, Sichuan University, Chengdu, China; ^2^Chinese Academy of Fiscal Sciences, Beijing, China; ^3^School of Management, Guangdong University of Technology, Guangzhou, China

**Keywords:** poverty experience, innovation performance, executive gender, market competition, R&D manipulation

## Abstract

This paper analyzes the impact mechanism of executive poverty experience on innovation performance from the two logics of “innate endowment” and “endogenous power.” It then explores the moderating role of executive characteristics, firm nature, and market competition from the perspective of heterogeneity, and finally proves the influence mechanism. Using the data of Chinese A-share listed companies from 2012 to 2020, the empirical results show that executives’ poverty experience improves corporate innovation performance. Further studies find that female executives with poverty experience have a more significant impact on innovation performance. Additionally, state-owned enterprises (SOEs) weaken the positive effects of executives with poverty experiences on innovation performance. The impact of executive poverty experience on innovation performance is more significant in fierce market competition. The mediating result suggests that executive poverty experience improves innovation performance partly by inhibiting R&D manipulation. The findings remain valid through Propensity Score Matching (PSM) tests, the Heckman two-stage, and alternative indicator measures. Using the early life poverty experience of executives, this study promotes research on the factors influencing corporate innovation. It also provides empirical evidence for improving corporate innovation performance through a study of moderating effects and influencing mechanisms.

## Introduction

The innovation has become a decisive factor in accelerating economic growth, profoundly affecting the competitive landscape of major nations, development of enterprises, and quality of life of people universally (Gherghina et al., 2020; Pan et al., 2022). The Chinese government attaches great importance to science and technology innovation and has achieved remarkable results. The World Intellectual Property Organization (WIPO) recently published its Global Innovation Index Report 2021, in which China ranked 12th, increasing steadily over the past 9 years. As the main force of innovation in the market economy, enterprises play an essential role in enhancing a country’s comprehensive innovation level and building an innovative nation (Gherghina et al., 2020). Consequently, understanding the internal rules of corporate innovation activities and studying their influencing factors have always been of interest to both theoretical and practical circles.

As decision-makers and senior executives of companies, executives’ background experiences influence corporate investment and financing decisions, as well as growth and development, etc. (Bernile et al., 2017; Bandiera et al., 2020; Park et al., 2021). Innovation is an essential strategy for sustainable corporate development, and executives with overseas, academic, and innovation experience have a positive impact on corporate innovation (Yuan and Wen, 2018; Lee J. M. et al., 2020; Quan et al., 2021).

Early life experiences of poverty can influence executives’ cognitive structure, decision-making preferences, value orientations, and so on (Holman and Silver, 1998; Pandya, 2020; O’Sullivan et al., 2021). This influence is “imprinted” as cognitive habits and behavioral characteristics, persistent in the subconscious of executives and reflected in their decisions (Marquis and Tilcsik, 2013). Executives’ early life experiences of poverty, such as lack of materials, lack of education, family financial difficulties, economic turmoil, and the surrounding poor environment, are more likely to shape their cognitive patterns, mental preferences, and value judgments later in life (Malmendier et al., 2011; Xu and Ma, 2021).

From a neuroscientific perspective, early life experiences of poverty have profound effects on the brain and biological systems. The traumatic impact on the brain is persistent and irreversible; and the early economic stress and upbringing of decision-makers continue to influence their psychological characteristics and financial behaviors (Adamkovič and Martončik, 2017; Ayllón and Fusco, 2017); Long et al. (2020) found executives who have experienced early life poverty, reduce the risk of stock price collapse.

Executives, as the core of management and strategic decision-making of listed companies, are more likely to be influenced by their early life poverty experiences in corporate decision-making, while corporate innovation is an important activity that concerns competitive market position and future sustainable development. Therefore, it is essential to study the impact of executives’ early life poverty experiences on corporate innovation performance. Firms and industries, as well as personal attributes, have significant effects on executive behavior. However, the extent to which poverty affects firm innovation performance and the boundaries of its effects are yet to be explored in related research. This study provides an opportunity to close this gap.

The research questions addressed in the present study are as follows: How does an executive’s early life experience of famine affect innovation performance and will the role of this poverty experience be affected by other factors? To solve these two problems, our research objectives include clarifying the theoretical logic of the impact of executive poverty experience on corporate behavior, analyzing the boundary conditions of the impact of senior executives’ poverty experience, discussing the interactive influence of internal and external characteristics of enterprises on enterprise investment, exploring the mechanism to improve the innovation performance of enterprises, and providing reference.

This study takes Chinese A-share listed companies from 2012 to 2020 as the research object and uses a multiple regression analysis method to analyze the relationship between executive poverty experience and enterprise innovation. Referring to the research of Long et al. (2020), we examine whether there is an early poverty experience in the childhood of senior executives by using whether they experienced the “Great Chinese Famine” from 1959 to 1961. The number of patents applied for by the enterprise within 3 years is used as the research variable for research innovation, and the number of patents granted within 3 years is used as the robustness test. Patents include invention, utility model, and design patents. Invention patents are more innovative than conventional patents. In this study, different patent types are regressed separately to understand the impact of executive poverty experience on various types of innovation.

**FIGURE 1:**
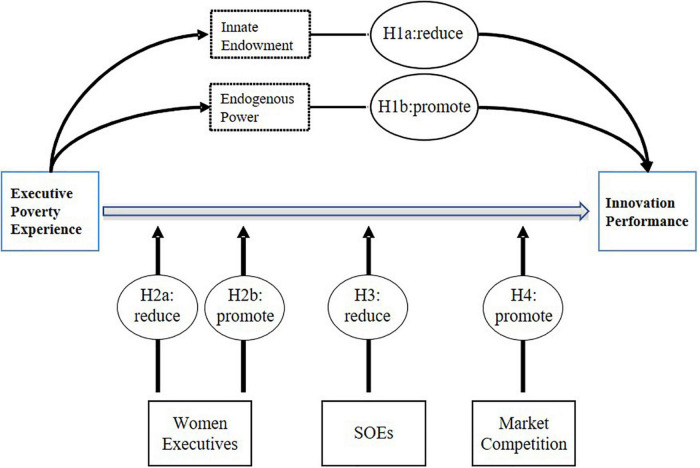
Conceptual framework.

This study adds scientific value by revealing the impact and mechanism of the early experience of senior executives on innovation from the perspective of poverty experience, and explores the boundary conditions of these experiences from many aspects, supplementing the relevant theories of senior management teams and enterprise innovation. Previous studies have explored the impact of executives on corporate decision-making based on the economic man hypothesis. This study found that executives with a higher moral level could inhibit R&D manipulation and improve innovation performance, deepening the cognition of corporate decision-making behavior motivation based on moral emotional factors. Thus, we analyze from the perspective of gender, property rights, and market competition to set a framework for the research hypothesis (see Figure 1) and extend the existing research on related topics.

The remainder of this paper is organized as follows. Section “Theoretical analysis and hypothesis development” reviews the relevant literature and proposes theoretical assumptions. Section “Data and methods” introduces the study data and methods. The results are presented in Section “Results.” Section “Discussion” discusses these findings. Finally, the conclusions, theoretical and practical implications, and current limitations are presented in Section “Conclusion.”

## Theoretical analysis and hypothesis development

Upper echelons theory suggests that innovation strategy choice, innovation outcomes, and innovation efficiency are influenced by executives’ limited rationality, cognitive patterns, and internal and external factors of the firm (Hambrick and Mason, 1984; Li Q. et al., 2018; You et al., 2020). To some extent, an executive’s personal experience will impact the distinctive knowledge structure, cognitive patterns, and value orientations in influencing their decision-making, feeding back to the firm, affecting strategic decision-making (Hambrick and Mason, 1984; Kong et al., 2021). When faced with opportunities to expand overseas, identify threats, and integrate resources, executives with poverty experiences are influenced by the subconscious “imprint” of their early life poverty experiences, which affects their risk appetite (Marquis and Tilcsik, 2013). In particular, when faced with risky activities such as innovation investment, the influence of early life experiences may be stimulated and amplified. Behavioral decisions may be characterized as “stigmatized” when faced with innovation activities. Therefore, this study analyzes the mechanism of poverty experiences on executives’ innovation decisions from the two logics of “innate endowment” and “endogenous power.”

First, from the perspective of “innate endowment,” the early stage of growth with fewer resources leads to a subconscious sense of material deprivation among executives, prone to attention depletion and risk aversion, and negatively impacts corporate innovation. Attention depletion refers to the fact that the experience of long-term poverty tends to cause individuals to pay more attention to immediate survival issues, obtain immediate benefits, and lack attention to long-term development issues (Haisley et al., 2008; Shah et al., 2012; Dalton et al., 2020; De Bruijn and Antonides, 2021). Therefore, individuals tend to focus more on immediate survival issues and less on long-term development issues. Innovation is characterized by high upfront investment costs and irreversibility of investment (Merton, 2013; Nanda and Rhodes-Kropf, 2017). If executives with poverty experience suffer from attention loss, they are likely to overlook the positive implications of innovation decisions on the company’s future development. In addition, early life experiences of poverty can limit human capital accumulation, and executives with a background in poverty may be more likely to feel insecure and risk-averse when faced with external changes (Lusardi and Mitchell, 2014; Bernile et al., 2017). Corporate innovation, as an activity with high uncertainty and large investment amounts, may deter executives from breaking the mold and taking innovation risks. The psychological effect of “innate endowment” will make executives with poverty experience easily form the thoughts of “focusing on the present” and “seeking stability”; their behavioral decision-making will be more stable. However, this has an adverse impact on corporate innovation performance.

Secondly, the logic of “endogenous power” suggests that the stress of early life poverty experiences can sharpen executives’ willpower and self-control, which in turn leads to the motivation to “change.” Poverty may lead executives to be mentally and voluntarily refined. Executives are more likely to form tenacious characters and action forces that are indomitable and never give up (Stephens et al., 2014). Executives who have experienced poverty during their youth are more likely to consider change when they are poor. Through their efforts at a later stage, they can change the poverty situation of their families, where “seeking change” becomes a meaningful way to change their predicament and a major characteristic of innovation. Born and raised in poverty, individuals becoming executives will have a process of cognitive reconstruction of risk, rethink the value of risk, re-perceive the size of risk, and find it easier to eliminate the fear of risk and uncertainty (Heilman et al., 2010). This results in stronger psychological tolerance and failure tolerance. Thus, these executives are more likely to choose innovative activities to promote the development of the enterprise when faced with innovative decisions. In this regard, executives with early life poverty experiences are more likely to devote more resources and energy to creative R&D activities.

Based on the above analysis, the following hypotheses are proposed:

H1a: Based on the logic of “innate endowment,” innovation performance is lower in firms with executives who have experienced poverty.

H1b: Based on the logic of “endogenous power,” innovation performance is higher in firms with executives who have experienced poverty.

According to upper echelons theory, executives of different genders can significantly differ in risk appetite and behavioral decisions (Hambrick and Mason, 1984; Liu, 2018; Post et al., 2022). Earlier studies found significant differences in investment decisions and risk preferences adopted by executives of different genders, with women exhibiting higher risk aversion and being less likely to be overconfident (Cumming et al., 2015; Chen et al., 2016). According to psychology, women’s emotional experiences are stronger and it is easier to perceive and identify external stimuli (Berkley, 1997). Consequently, women are prone to be nervous about risks (Maxfield et al., 2010). Based on conservative and robust traits, female executives are more willing to improve corporate disclosure (Gul et al., 2011); Faccio et al. (2016) analyzed European listed companies and found that female CEOs take 2% less risk than male CEOs, demonstrating that female CEOs exhibit more robust behaviors. Thus, when female executives experience poverty, “innate endowments,” and “gender differences,” may result in stronger risk-averse preferences and conservative business and investment decisions in high-risk innovation activities, possibly reducing company innovation performance.

However, women are at a disadvantage in the workplace; they need to work harder, and have a longer-term and innovative strategy to overcome gender biases in the workplace to gain promotion (Wille et al., 2018). For conservative and steady women, it is challenging to assume the role of corporate executives and lead their companies to success. In the context of the poverty experience, women need to be more decisive, independent, and hard working to thrive in the workplace in the long-term. Moreover, in the field of innovation, it takes courage to take risks and the ability to deal with them. Female executives possess unique human capital, such as attentiveness and sensitivity, and such attributes may help female executives grasp changes in customers and markets, which are important drivers for corporate innovation (Torchia et al., 2018; Audretsch et al., 2022). Finally, female executives are more likely to adopt a democratic approach and have greater influence, which can increase organizational members’ motivation to participate in decision-making (Gul et al., 2011; Carbajal, 2018). A tolerant and democratic attitude also contributes to a favorable innovation climate in the company, which drives the technological innovation process. Accordingly, when female executives have experienced poverty, they may abandon traditional female risk preferences and reinforce the logical path of “endogenous power,” having a positive impact on the innovation performance of the company.

Based on the above analysis, the following hypotheses are proposed:

H2a: Female executives suppress the positive impact of poverty experiences on firm innovation performance.

H2b: Female executives promote a positive effect of poverty experience on firm innovation performance.

State-owned and private enterprises differ significantly in terms of corporate governance, operational objectives, investment decisions, and so on, which are likely to affect the motivation and costs associated with innovation decisions (Belloc, 2014; Jia et al., 2019). State owned enterprises (SOEs) generally face burdens such as redundant employee costs, high tax rates, and policy investments, which crowd out significant corporate economic resources and adversely affect innovation activities that require them. Additionally, SOEs implement annual and tenure appraisal systems; where an important indicator of the evaluation of senior management is the level of value maintenance and appreciation of state-owned assets, directly affect the political advancement of senior management (Bai and Bennington, 2005). However, innovative investment activities are characterized by high investment and high-risk. Once the investment fails, the profit level and asset situation of the enterprise fluctuate (Hock-Doepgen et al., 2021), which has a negative impact on the performance appraisal of executives. In cases where the operational pressure of SOEs conflicts with innovation risk-taking, executives with poverty experience are more likely to adopt a strategy of stable operations and reduce corporate innovation activity. Therefore, SOEs undermine the “endogenous power” logic of the impact of executives with poverty experience on innovation. In situations of conflict between innovation risk-taking and the operating pressures of SOEs, executives with poverty experience are more likely to adopt a strategy of stable operation and reduce corporate innovation activities.

Based on the above analysis, the following hypothesis is proposed.

H3: SOEs significantly inhibit the positive effects of executives with poverty experiences on innovation performance.

Competitive market pressure is an exogenous driving force for firms to innovate, assisting them to gain competitive advantage in the market. Competitive market pressure can also influence executives’ innovation behavior. Firms face increased business risks in a competitive market environment, and innovation assists in gaining a certain monopoly market and excess profits through R&D patents (Boone, 2001). Andrevski and Ferrier (2019) found that excessive competition leads to higher costs and lower performance; however, when firms have special technological resources, they can benefit from competition. Therefore, in the face of fierce market competition, corporate executives have strong incentives to conduct R&D innovation activities based on salary contracts, occupational safety, and professional reputation. For executives with poor experience, perceived market or business risk is greater when the company faces a higher degree of external competition. Therefore, from the logical perspective of “endogenous power,” in a situation of intense market competition, executives with poor experience are more motivated to proactively promote corporate innovation, thereby acquiring innovation benefits, and gaining advantages in market competition. However, when market competition is weak, corporate innovation gains are insufficient. Therefore, executives cannot be encouraged to carry out innovation activities.

Based on the above analysis, the following hypothesis is proposed.

H4: The degree of market competition significantly increases the positive effect of having poverty-experienced executives on innovation performance.

## Data and methods

### Sample selection

This study focuses on the innovation performance of a full sample of companies and selects A-share listed companies from 2012 to 2020 as the research object. The following treatments were carried out during the data collection process. We excluded financial industry code companies, including banks and non-banking financial enterprises, insolvent companies, ST and *ST companies, and samples with missing data. The tails of the key continuous variables were reduced by 1% above and below. Ultimately, 20,834 observations were obtained. The executive background, corporate characteristics, and other data are taken from the China Stock Market Accounting Research Database (CSMAR) and China Research Data Services Platform (CNRDS). The statistical software used in this study is STATA 15.0.

### Variable measures

#### Explanatory variables

We examined general managers responsible for making and executing business decisions. There are two methods to measure the early life poverty experience of senior executives: one is whether senior executives were born in a poor national county, and the other is whether senior executives experienced economic difficulties in their childhood, such as 1929–1933 in the United States and 1959–1961 in China (Long et al., 2020). Information disclosure of senior executives in listed companies has less disclosed information on the origin of senior executives, especially at the county level. Further, senior executives seldom publicly mention their place of birth. Thus, there are many missing values in collecting whether senior executives were born in poor counties, regardless of whether the information was from the company’s annual report or online. However, the corporate annual report discloses the age information of senior executives, which can be used to obtain complete data on whether senior executives have experienced poverty. Therefore, the early life poverty experience is determined by whether senior executives experienced the “Great Chinese Famine” from 1959 to 1961. With regard to the definition of childhood, the upper limit of the child time limit is roughly defined as 14 years old, regardless of the use of age, brain development, or psychological maturity indicators (Tulving, 2002). This study selected the period of 0–14 years as the time span for childhood, considering the continuity of psychological development. The Famine is assigned a value of 1 when the executive was born between 1947 and 1961, meaning that the executive’s childhood was from 1959 to 1961; otherwise, the value is 0.

#### Explained variables

The variables used to measure the innovation performance of enterprises are typically the number of patent applications or patents granted to enterprises. However, the period for granting patents cannot be unified effectively because the time from application to grant varies, with invention patents taking longer to grant (generally 2–3 years) and other patents taking shorter. Therefore, the number of patents applied for by enterprises is used as the research variable, and the number of patents granted within 3 years is used as a robustness test. There are three main types of patents: invention, utility model, and design. Different patents differ significantly in their innovativeness and invention patents are generally considered more innovative. We use three different measurement methods to measure the innovation performance of enterprises. The variable SLpatent1 is calculated as the natural logarithm of the sum of all patent applications; the variable Lpatent2 is calculated as the natural logarithm of the number of invention patent applications; and the variable Lpatent3 is calculated as the natural logarithm of the sum of the number of utility model patents and design patent applications, respectively.

#### Moderating variables

This study selected three moderating variables from the perspective of heterogeneity to test the boundary effect of the executive poverty experience: executive gender, the nature of property rights, and the degree of market competition. First, the variable Female is set, and Female has a value of 1 if the executive is female, and 0 otherwise. In the next step, the variable Soe is set to have a value of 1 when the executive’s company is a SOE and 0 if it is a private enterprise. In this study, we evaluate whether it is a SOE based on the top ten shareholders of the enterprise, and define it as a SOE if the largest shareholder of the company is the state or national legal entity. The third step involves setting the variable CompH and measuring the degree of marketplace competition using the Lerner Index. To calculate each company’s Lerner index, the specific formula is PCM = (operating income operating costs–selling expenses–administrative expenses)/operating income. The industry average of the Lerner index calculated above is reduced to obtain a measure of competitive impact; that is, the Lerner index of a single listed company is subtracted from the sales-weighted Lerner index within the industry. The larger the CompH variable, the higher the competitive position in the industry and the lower the degree of competition; the smaller the CompH variable, the higher the competitive pressure faced by the company. The regulatory variable is multiplied by the independent variable because of the regression, and the magnitude, significance, and positive and negative signs of the multiplicative term illustrate the extent of the regulatory effect. Since the independent variable is a 0–1 variable, it is easy for the interactive term to produce strong collinearity. Therefore, in the regulatory effect model, the independent and regulatory variables are centralized (i.e., the independent variable minus the sample mean), and the interactive term is generated for multiple regression analysis.

#### Control variables

Some firm-specific characteristics and fixed effects may affect innovation performance and thus should be controlled. Previous studies indicate that innovation performance is more likely to be created in firms with lower leverage ratios, higher profitability, more total assets, longer listing durations, more intangible assets, or more free cash flow (Acharya and Xu, 2017; Petruzzelli et al., 2018; Xin et al., 2019b). Previous studies also indicate that sales growth, cash holdings, ownership concentration, and management shareholding influence innovation performance (Belloc, 2012; De Jong et al., 2014; Yang et al., 2015; Xin et al., 2019a; Lee C. et al., 2020; Zhang et al., 2021).

Consistent with prior research, this study controls for the following factors that may impact firms’ innovation performance. The leverage ratio is measured as the ratio of total debt to total assets (Xin et al., 2019b). Firm profitability is measured using return on assets (ROA) (Acharya and Xu, 2017). Firm growth is a proxy for the sample firm’s market performance and is measured as current sales revenue minus previous sales revenue, divided by current sales revenue (De Jong et al., 2014). Firm size is measured by log-transformed total assets at the year-end (Petruzzelli et al., 2018). The firm’s listing age is measured by the log-transformed (one plus) listing duration (Petruzzelli et al., 2018). Tangible assets are fixed assets scaled by total assets (Acharya and Xu, 2017). Equity concentration controlled for the sample firm’s shareholders dominates and is measured by the percentage of shares owned by the largest shareholder (Belloc, 2012; Yang et al., 2015). Free cash flow is measured as the ratio of a company’s cash flow from operating activities to total assets (Xin et al., 2019a). Cash holdings are measured using cash and cash equivalents scaled by total assets (Acharya and Xu, 2017). Management shareholding is measured as total management shareholding divided by the total number of shares (Zhang et al., 2021). Additionally, the regression model includes industry and year dummy variables, which can be used to control for the impact of industry and year (Zhang et al., 2021). Table 1 presents the variable names, symbols, and definitions for all the variables.

**TABLE 1:** Variable definitions.

Nature of variables	Variable name	Variable symbols	Variable definition
Dependent variable	Corporate innovation performance	SLpatent1	Natural logarithm of the number of invention, utility and design patent applications of the company
		Lpatent2	Natural logarithm of the number of invention patent applications of the company
		Lpatent3	Natural logarithm of the sum of the number of utility and design patent applications for the company
Independent variable	Executive poverty experience	Famine	The value of this indicator is 1 if the executive has experienced the “3 Years of Difficulty Period” as a child, i.e., was born between 1947 and 1961. Otherwise, it is 0.
Adjustment variables	Executive gender	Female	Female is assigned a value of 1, or 0 otherwise.
	Property rights	Soe	State-owned enterprises take the value of 1 for this indicator, while private enterprises take the value of 0
	Market competition	CompH	The Lerner Index, adjusted for industry
Control variables	Financial leverage	Leve	leverage ratio, i.e., total liabilities divided by total assets
	Profitability	Roa	Net profit margin on total assets, i.e., the ratio of a company’s net profit to its total assets
	Firm growth	Growth	Current period sales revenue minus prior period sales revenue, divided by current period sales revenue
	Firm size	Size	Natural logarithm of total assets at the end of the year
	Firm age	Listage	Natural logarithm of the number of years the company has been listed
	Size of fixed assets	Ppeta	Ratio of fixed assets to total assets
	Shareholding Concentration	Share1	Percentage of shareholding of the largest shareholder
	Free cash flow	Cfo	Ratio of cash flow from operating activities to total assets of the company
	Cash holding levels	Cash	Ratio of the company’s cash and cash equivalents to the company’s total assets
	Management shareholding	Esh	Total number of shares held by management divided by total number of shares in the company
	Industry dummy variables	Indu	Following the guidelines of the Industry Classification of Listed Companies (2012 Edition) of the Securities and Futures Commission, the manufacturing industry is classified according to the secondary industry code, while the remaining sector is classified according to the primary industry code.
	Year dummy variables	Year	The study sample covered a period of 19 years, and 18 dummy variables were generated.

### Descriptive statistics

Table 2 presents the descriptive statistics of the variables. The mean value of SLpatent1 is 0.791, the median is 0, and the maximum value is 9.724. The mean value of Lpatent2 is 0.613 and the maximum value is 8.996. The mean value of Lpatent3 is 0.483 and the maximum value is 9.198. The mean value of the independent variable Famine is 0.186, indicating that the percentage of executives who experienced the Great Chinese Famine in the sample is 18.6%. The descriptive statistics of the moderating variables show that the proportion of female executives in China’s listed companies is very low, accounting for only 6.8%. SOEs accounted for 31.4% of the sample, and the average Lerner index is 0.022. The descriptive statistics of the other control variables are similar to those in related studies.

**TABLE 2:** Descriptive statistics.

Variables	Average value	Standard deviation	Minimum value	25th percentile	Median	75th percentile	Maximum value
SLpatent1	0.791	1.380	0.000	0.000	0.000	1.099	9.724
Lpatent2	0.613	1.165	0.000	0.000	0.000	0.693	8.996
Lpatent3	0.483	1.111	0.000	0.000	0.000	0.000	9.198
Famine	0.186	0.389	0.000	0.000	0.000	0.000	1.000
Female	0.068	0.251	0.000	0.000	0.000	0.000	1.000
SOE	0.314	0.464	0.000	0.000	0.000	1.000	1.000
CompH	0.022	0.136	−0.824	−0.043	0.014	0.085	0.551
Leve	0.410	0.203	0.053	0.244	0.398	0.560	0.881
Roa	0.041	0.059	−0.244	0.016	0.039	0.070	0.193
Growth	0.187	0.658	−0.848	−0.049	0.092	0.260	4.802
Size	22.143	1.276	19.902	21.218	21.960	22.851	26.179
Listage	1.985	0.927	0.000	1.386	2.197	2.833	3.296
Ppeta	0.209	0.158	0.002	0.087	0.177	0.296	0.695
Share1	0.344	0.146	0.087	0.231	0.324	0.441	0.743
Cfo	0.049	0.067	−0.149	0.011	0.048	0.089	0.239
Cash	0.164	0.126	0.012	0.074	0.128	0.216	0.609
Esh	0.153	0.205	0.000	0.000	0.016	0.298	0.685

## Results

### Correlation analysis

Table 3 provides the Pearson correlation coefficient matrix of key variables. The results show that the correlation coefficient between SLpatent1 and Lpatent2 is 0.956, and the correlation coefficient between Lpatent2 and Lpatent3 is 0.899. The correlation coefficient between Lpatent2 and Lpatent3 is 0.784. These results indicate that the correlation of the three alternative variables of innovation performance is very high, consistent with the index setting. The correlation coefficient of the independent variable Famine and SLpatent1 is 0.019, the correlation coefficient of the independent variable Famine and Lpatent2 is 0.013, and the correlation coefficient of Famine and Lpatent3 is 0.032. Famine is significantly and positively correlated with SLpatent1 and Lpatent3; however, has weaker significance with Lpatent2. Without controlling for other factors, these results suggest that executives who have experienced the Great Chinese Famine have a significant positive correlation with corporate innovation performance, which to some extent supports the previous hypothesis. According to Table 3, there is no significant multicollinearity in the study variables. In support of this conclusion, the highest off-table VIF value is only 2.05.

**TABLE 3:** Correlation coefficient matrix.

Variables	SLpatent1	Lpatent2	Lpatent3	Famine	Female	SOE	CompH	Leve
Lpatent2	0.956***	1						
Lpatent3	0.899***	0.784***	1					
Famine	0.019***	0.013*	0.032***	1				
Female	−0.030***	−0.031***	−0.025***	0.006	1			
SOE	0.129***	0.128***	0.125***	0.035***	−0.065***	1		
CompH	0.021***	0.024***	0.000	−0.019***	0.020***	−0.146***	1	−
Leve	0.138***	0.129***	0.149***	0.023***	−0.020***	0.308***	−0.285***	1
Roa	0.024***	0.026***	0.006	0.007	0.007	−0.118***	0.562***	−0.366***
Growth	0.021***	0.019***	0.014**	−0.022***	−0.003	−0.046***	0.088***	0.063***
Size	0.387***	0.379***	0.362***	0.025***	−0.039***	0.373***	0.009	0.539***
Listage	0.137***	0.136***	0.113***	0.001	−0.021***	0.448***	−0.252***	0.402***
Ppeta	0.022***	0.022***	0.023***	0.062***	−0.031***	0.215***	−0.110***	0.101***
Share1	0.048***	0.044***	0.068***	0.038***	0.008	0.232***	0.052***	0.050***
Cfo	0.040***	0.043***	0.028***	0.009	0.017**	−0.029***	0.301***	−0.172***
Cash	−0.082***	−0.075***	−0.078***	0.005	0.031***	−0.089***	0.178***	−0.406***
Esh	−0.104***	−0.105***	−0.094***	−0.046***	0.041***	−0.479***	0.196***	−0.339***
**Variables**	**Roa**	**Growth**	**Size**	**Listage**	**Ppeta**	**Share1**	**Cfo**	**Esh**
Growth	0.135***	1						
Size	−0.045***	0.079***	1					
Listage	−0.247***	−0.034***	0.451***	1				
Ppeta	−0.088***	−0.047***	0.120***	0.153***	1			
Share1	0.123***	0.009	0.174***	−0.071***	0.090***	1		
Cfo	0.390***	0.022***	0.061***	−0.028***	0.214***	0.094***	1	
Cash	0.268***	−0.035***	−0.264***	−0.280***	−0.318***	0.035***	0.162***	1
Esh	0.186***	0.009	−0.381***	−0.561***	−0.179***	−0.090***	0.021***	0.185***

*p < 0.1, **p < 0.05, ***p < 0.01, same later.

### Empirical regression results

Table 4 shows the impact of executive poverty experiences on enterprise innovation performance. Columns (1–3) show the univariate regression results of the independent variable Famine on the dependent variables SLpatent1, Lpatent2, and Lpatent3, respectively. The findings indicate that the coefficients of the effect of the independent variable Famine on the dependent variable are significantly positive, regardless of other factors. Columns (4–6) show the regression results after adding control variables. The regression coefficients of Famine with SLpatent1, Lpatent2, and Lpatent3 are 0.056, 0.036, and 0.068, respectively, which are significantly positive at the 5, 10, and 1% levels, respectively. These results indicate that experiencing the Great Chinese Famine during childhood significantly enhances the overall level and specific types of innovation in firms. Results in Table 4 support the previous logical perspective of “endogenous power,” (i.e., H1b) which suggests that executives who have experienced poverty are more likely to enhance their competitive advantage through innovation and improve their innovation performance to a greater extent. Regarding the control variables, corporate financial leverage (Leve), sales growth (Growth), and fixed asset size (Ppeta) significantly lower the level of innovation, while corporate profitability (ROA), firm size (Size), and firm age (Listage) significantly increase innovation performance.

**TABLE 4:** Regression results of executive poverty experience and firm innovation performance.

Variables	(1)	(2)	(3)	(4)	(5)	(6)
	SLpatent1	Lpatent2	Lpatent3	SLpatent1	Lpatent2	Lpatent3
Famine	0.068***	0.040*	0.092***	0.056**	0.036*	0.068***
	(2.627)	(1.865)	(4.256)	(2.532)	(1.933)	(3.614)
Leve				−0.382***	−0.344***	−0.211***
				(−6.908)	(−7.281)	(−4.746)
Roa				0.283*	0.244*	−0.041
				(1.715)	(1.748)	(−0.306)
Growth				−0.030**	−0.025**	−0.033***
				(−2.398)	(−2.305)	(−3.306)
Size				0.525***	0.438***	0.381***
				(47.094)	(43.371)	(37.953)
Listage				0.039***	0.029***	0.003
				(3.291)	(2.875)	(0.354)
Ppeta				−0.587***	−0.459***	−0.367***
				(−8.655)	(−7.939)	(−6.552)
Share1				0.028	0.019	0.105*
				(0.412)	(0.333)	(1.873)
Cfo				0.054	−0.003	0.130
				(0.402)	(−0.024)	(1.200)
Cash				0.020	0.038	0.058
				(0.271)	(0.613)	(0.985)
Esh				0.019	−0.014	0.042
				(0.387)	(−0.351)	(1.060)
Indu	No	No	No	Yes	Yes	Yes
Year	No	No	No	Yes	Yes	Yes
Constant	0.779***	0.606***	0.466***	−10.705***	−8.910***	−8.008***
	(74.673)	(68.338)	(56.324)	(−45.452)	(−41.709)	(−38.123)
*N*	20834	20834	20834	20834	20834	20834
Adj-*R*^2^	0.000	0.000	0.001	0.236	0.221	0.211

T-values in parentheses, *p < 0.1, **p < 0.05, ***p < 0.01, same later.

The effect of executive gender heterogeneity on the impact of executive poverty on corporate innovation is examined empirically based on executive gender heterogeneity. We incorporated the interaction terms of the variables Famine and Female into the regression model. The regression results are presented in Table 5, showing that the interaction terms are significantly positive. It is evident from the results that the positive impact of poverty experiences on innovation is enhanced when executives are female. Female executives have a relatively weaker impact on invention patents and a more significant impact on utility model and design patents. This interesting finding differs from the traditional conclusion that women are conservative and steady. The study argues that female executives who have experienced poverty are likely to demonstrate a greater risk appetite and will be more willing to take risks and carry out innovative activities. Our study complements and enhances the existing literature on women.

**TABLE 5:** Regression results of moderating effects under gender heterogeneity.

Variables	(1)	(2)	(3)
	SLpatent1	Lpatent2	Lpatent3
Famine*Female	0.219**	0.157**	0.234***
	(2.420)	(2.041)	(2.941)
Famine	0.041*	0.025	0.051***
	(1.787)	(1.309)	(2.665)
Female	−0.049	−0.044	−0.042
	(−1.349)	(−1.402)	(−1.475)
Leve	−0.383***	−0.344***	−0.211***
	(−6.912)	(−7.284)	(−4.754)
Roa	0.278*	0.240*	−0.046
	(1.683)	(1.720)	(−0.347)
Growth	−0.030**	−0.025**	−0.033***
	(−2.357)	(−2.272)	(−3.250)
Size	0.524***	0.438***	0.381***
	(47.115)	(43.360)	(37.992)
Listage	0.038***	0.029***	0.002
	(3.217)	(2.815)	(0.254)
Ppeta	−0.587***	−0.459***	−0.367***
	(−8.658)	(−7.939)	(−6.556)
Share1	0.032	0.023	0.110*
	(0.479)	(0.393)	(1.958)
Cfo	0.056	−0.000	0.131
	(0.418)	(−0.004)	(1.213)
Cash	0.016	0.036	0.053
	(0.213)	(0.566)	(0.907)
Esh	0.014	−0.018	0.036
	(0.282)	(−0.430)	(0.905)
Indu	Yes	Yes	Yes
Year	Yes	Yes	Yes
Constant	−10.687***	−8.897***	−7.990***
	(−45.454)	(−41.685)	(−38.154)
*N*	20834	20834	20834
Adj-*R*^2^	0.237	0.221	0.212

T-values in parentheses, *p < 0.1, **p < 0.05, ***p < 0.01.

Additionally, based on the corporate property rights and market competition perspectives, the boundary effects of executive poverty experience on the impact of corporate innovation are tested empirically, and the regression results are shown in Table 6. Columns (1–3) in Table 6 provide the regression results for the moderating effect of property rights. In the regression with the dependent variables SLpatent1 and Lpatent2, the coefficients of the interaction term Famine*Soe are −0.094 and −0.113, respectively, which are statistically significant at the 10 and 1% levels, respectively. Nevertheless, it is insignificant in the regressions where Lpatent3 is the dependent variable. In SOEs, executives with poverty experience contribute less to innovation; while executives with poverty experiences have a greater impact on innovation in non-state-owned enterprises. Thus, Hypothesis 3 is verified. More specifically, property rights have a greater impact on innovative invention patents and do not affect utility models or design patents. One possible reason is that the risks in these two types of innovation are small, and executives of SOEs prefer to increase their innovation levels through these two types of patents to meet the innovation requirements of their performance appraisals. As a result, the poverty experience will not be affected.

**TABLE 6:** Regression results of the moderating effects of the nature of property rights and market competition.

Variables	(1)	(2)	(3)	(4)	(5)	(6)
	SLpatent1	Lpatent2	Lpatent3	SLpatent1	Lpatent2	Lpatent3
Famine*Soe	−0.094*	−0.113***	0.008			
	(−1.939)	(−2.768)	(0.202)			
Famine*CompH				−0.288**	−0.189*	−0.292***
				(−2.278)	(−1.811)	(−2.859)
Famine	0.060***	0.040**	0.069***	0.061***	0.039**	0.073***
	(2.723)	(2.172)	(3.703)	(2.757)	(2.114)	(3.872)
Soe	0.143***	0.133***	0.086***			
	(6.052)	(6.516)	(4.405)			
CompH				−0.054**	−0.035*	−0.055***
				(−2.304)	(−1.827)	(−2.915)
Leve	−0.406***	−0.365***	−0.225***	−0.385***	−0.345***	−0.213***
	(−7.292)	(−7.693)	(−5.024)	(−6.952)	(−7.313)	(−4.808)
Roa	0.258	0.221	−0.056	0.353**	0.289**	0.032
	(1.558)	(1.578)	(−0.423)	(2.104)	(2.042)	(0.236)
Growth	−0.026**	−0.021*	−0.031***	−0.030**	−0.025**	−0.033***
	(−2.070)	(−1.953)	(−3.049)	(−2.392)	(−2.301)	(−3.298)
Size	0.520***	0.434***	0.378***	0.525***	0.438***	0.381***
	(46.703)	(43.052)	(37.679)	(47.126)	(43.388)	(37.986)
Listage	0.021*	0.012	−0.008	0.039***	0.029***	0.003
	(1.680)	(1.149)	(−0.759)	(3.237)	(2.834)	(0.292)
Ppeta	−0.644***	−0.512***	−0.400***	−0.591***	−0.461***	−0.371***
	(−9.420)	(−8.792)	(−7.093)	(−8.712)	(−7.978)	(−6.621)
Share1	−0.048	−0.050	0.059	0.023	0.016	0.101*
	(−0.704)	(−0.856)	(1.042)	(0.348)	(0.286)	(1.794)
Cfo	0.134	0.073	0.174	0.071	0.008	0.148
	(1.001)	(0.651)	(1.605)	(0.527)	(0.073)	(1.359)
Cash	−0.047	−0.024	0.018	0.019	0.038	0.057
	(−0.641)	(−0.383)	(0.312)	(0.260)	(0.605)	(0.960)
Esh	0.097*	0.058	0.087**	0.020	−0.014	0.042
	(1.921)	(1.401)	(2.155)	(0.395)	(−0.344)	(1.067)
Indu	Yes	Yes	Yes	Yes	Yes	Yes
Year	Yes	Yes	Yes	Yes	Yes	Yes
Constant	−10.534***	−8.761***	−7.885***	−10.705***	−8.910***	−8.006***
	(−44.511)	(−40.921)	(−37.467)	(−45.476)	(−41.722)	(−38.148)
*N*	20834	20834	20834	20834	20834	20834
Adj-*R*^2^	0.238	0.223	0.212	0.236	0.221	0.211

T-values in parentheses, *p < 0.1, **p < 0.05, ***p < 0.01.

Columns (4–6) of Table 6 present the regression results for the moderating effect of market competition. The interaction terms Famine × CompH were both significantly negative in the regression results. Since the variable CompH is the industry-adjusted Lerner index, the smaller the value, the greater is the competitive pressure. The negative interaction coefficient indicates that the higher the competitive pressure faced by the firm, the greater the impact of the executive poverty experience on firm innovation. In other words, the executive poverty experience has a significant impact on corporate innovation under fierce market competition. Thus, H4 is verified. The coefficients of the interaction term Famine*CompH are −0.288, −0.189 and −0.292, respectively, which are statistically significant at 5, 10, and 1%, respectively, when the dependent variables are SLpatent1, Lpatent2, and Lpatent3.

### Impact mechanisms

The above theoretical analysis and empirical tests have confirmed that executives with poverty experiences have a greater positive impact on firms’ innovation performance. In this section, we analyze the impact mechanism from the perspective of R&D manipulation. R&D investment is the main driving force and engine of firm innovation; however, due to the professional and technical nature of R&D activities, there is a high degree of information asymmetry in the R&D process. It is possible that some executives whitewash R&D investment through surplus manipulation to satisfy performance appraisals, obtain personal gains, or obtain government subsidies, adversely affecting corporate innovation. If executives possess high ethical integrity, they can avoid compromising their business decisions with short-term goals, resist egoistic and opportunistic tendencies, and inhibit R&D manipulation.

Research has demonstrated that childhood experiences contribute to the development of an individual’s character and will continue to influence moral judgment and value orientation. Early life experiences of poverty have a stronger impact on a CEO and are more likely to develop a sense of social responsibility and morality, such as greater compassion (Malmendier et al., 2011) and an increased sense of responsibility (O’Sullivan et al., 2021; Xu and Ma, 2021). According to Feng and Johansson (2018), CEOs who experienced the Great Chinese Famine in their youth were less likely to commit fraud and showed higher levels of ethics (Feng and Johansson, 2018). Therefore, executives with early life poverty experiences are more likely to restrain R&D manipulation and improve innovation performance.

Based on the above analysis, this study proposed a mediating effect test. In accordance with Gunny’s method (Gunny, 2010), variable *Abn* measures the level of corporate R&D manipulation. In this model, normal R&D expenditure is estimated using Equations (1) and (2), and abnormal expenditure is calculated using Equation (3). In the following equations, *Rd* is the firm’s R&D expenditure, *Ta* is total assets, *Mv* is the natural logarithm of the firm’s market value, *Tbq* is Tobin’s *Q*-value, and *Int* is the firm’s operating profit.


$$\frac{R\,{\text{d}}_{i,t}}{T\,a_{i,t-1}}=\beta \,0+\beta \,1\,\frac{1}{T\,a_{i,t-1}}+\beta \,2\,M\,v_{i,t}+\beta \,3\,T\,b\,p_{i,t}$$

(1) $$+\beta \,4\,\frac{I\,n\,t_{i,t}}{T\,a_{i,t-1}}+\beta \,5\,\frac{R\,d_{i,t-1}}{T\,a_{i,t-1}}+\varepsilon_{i,t}$$

$$N\,{\text{orm}}_{i,t}=\overset{\wedge }{\beta \,0}+\overset{⌢}{\beta }1\,\frac{1}{T\,a_{i,t-1}}+\overset{⌢}{\beta }2\,M\,v_{i,t}+\overset{⌢}{\beta }3\,T\,b\,p_{i,t}$$

(2) $$+\overset{⌢}{\beta }4\frac{I\,n\,t_{i,t}}{T\,a_{i,t-1}}+\overset{⌢}{\beta }5\frac{R\,d_{i,t-1}}{T\,a_{i,t-1}}$$

(3) $$A\,b\,n_{i,t}=\frac{R\,d_{i,t}}{T\,a_{i,t-1}}-N\,o\,r\,m_{i,t}$$

This study first examines the effect of the independent variable Famine on R&D manipulation (*Abn*) and then applies *Abn* to the regression model as a mediating variable to verify the significance of the independent and mediating variables. The regression results are presented in Table 7. Column (1) of Table 7 examines the relationship between executive poverty experience and R&D manipulation when Famine is the independent variable. The regression coefficient is 0.001, which is significant at the 1% level. This indicates that executives’ early life poverty experience significantly reduces the level of corporate R&D manipulation. According to the regression results in Column (2), the effect of the independent variable Famine on innovation performance remains significantly positive, whereas the effect of the mediating variable *Abn* on overall firm innovation performance (SLpatent1) is significantly negative. In other words, the mediating effect persists for executives’ early life poverty experience by reducing R&D manipulation, which in turn enhances the firm’s performance in innovation. Furthermore, when analyzing the impact of different types of innovation, the results in Columns (3) and (4) indicate that the effect of R&D manipulation on the number of patent applications (Lpatent2) is negative and statistically significant at the 10% level (coefficient = −2.015, *t*-value = −1.66). The effect of R&D manipulation on the number of types and design patent applications (Lpatent3) is significantly negative at the 1% level (coefficient = −4.247, *t*-value = −3.937). Therefore, this result suggests that the mediating effect of R&D manipulation is more pronounced on the impact of poverty-experienced executives in the utility model and design patents, and relatively weak in more innovative invention patents.

**TABLE 7:** Test for mediating effects of R&D manipulation.

Variables	(1)	(2)	(3)	(4)
	Abn	SLpatent1	Lpatent2	Lpatent3
Famine	−0.001***	0.054**	0.035*	0.066***
	(−5.352)	(2.455)	(1.876)	(3.495)
Abn		−3.210**	−2.015*	−4.247***
		(−2.329)	(−1.660)	(−3.937)
Leve	0.001***	−0.378***	−0.341***	−0.204***
	(4.475)	(−6.825)	(−7.219)	(−4.614)
Roa	0.010***	0.316*	0.265*	0.003
	(9.538)	(1.910)	(1.893)	(0.021)
Growth	0.001***	−0.027**	−0.023**	−0.029***
	(9.061)	(−2.138)	(−2.115)	(−2.880)
Size	0.000	0.525***	0.438***	0.381***
	(0.510)	(47.135)	(43.397)	(38.005)
Listage	0.000***	0.040***	0.030***	0.004
	(3.782)	(3.353)	(2.922)	(0.455)
Ppeta	−0.003***	−0.596***	−0.464***	−0.379***
	(−7.767)	(−8.774)	(−8.020)	(−6.753)
Share1	−0.000	0.027	0.019	0.105*
	(−0.068)	(0.411)	(0.332)	(1.873)
Cfo	0.004***	0.066	0.005	0.147
	(4.647)	(0.496)	(0.046)	(1.354)
Cash	0.002***	0.026	0.042	0.066
	(4.051)	(0.353)	(0.673)	(1.119)
Esh	−0.000	0.019	−0.015	0.041
	(−0.610)	(0.375)	(−0.360)	(1.039)
Indu	Yes	Yes	Yes	Yes
Year	Yes	Yes	Yes	Yes
Constant	0.008***	−10.680***	−8.894***	−7.976***
	(7.237)	(−45.396)	(−41.676)	(−38.077)
N	20834	20834	20834	20834
Adj-*R*^2^	0.132	0.237	0.221	0.212

T-values in parentheses, *p < 0.1, **p < 0.05, ***p < 0.01.

### Robustness tests

#### Propensity score matching

Since an executive’s early life poverty experience is innate, there is no reverse causality issue in terms of the firm’s impact. However, this study focuses on all A-share listed companies, and the percentage of executives with early life poverty experiences is only 18.6%, suggesting that the sample selection may have been biased. Therefore, the propensity matching score method (PSM) is used to conduct the robustness test. The overall sample is first divided into an experimental group of executives who have experienced the Great Chinese Famine and a control group of executives who have not experienced the Great Chinese Famine. Second, a logit model is used to estimate the propensity scores of executives who have experienced the Great Chinese Famine. The dependent variable is poverty experience (Famine), which includes financial leverage (Leve), profitability (Roa), firm growth (Growth), firm size (Size), firm age (Listage), fixed assets share (Ppeta), equity concentration (Share1), free cash flow (Cfo), cash holding level (Cash), and management ownership (Esh) while controlling for industry and year fixed effects. Third, the one-to-one nearest neighbor matching method is used for matching, and 7081 observations were obtained after matching. By matching the samples, the standard deviations of the experimental and control groups were effectively controlled, and the standard deviation of the majority of samples decreased by more than 80%. The sample regression results are shown in Columns (1–3) of Table 8, and the previous conclusions are still valid when the sample selection deviation is excluded.

**TABLE 8:** PSM test and Heckman two-stage test regression results.

Variables	(1)	(2)	(3)	(4)	(5)	(6)
	SLpatent1	Lpatent2	Lpatent3	SLpatent1	Lpatent2	Lpatent3
Famine	0.074**	0.057**	0.078***	0.061***	0.041**	0.071***
	(2.556)	(2.349)	(3.211)	(2.738)	(2.185)	(3.748)
Leve	−0.632***	−0.546***	−0.431***	−0.397***	−0.356***	−0.214***
	(−6.274)	(−6.466)	(−5.106)	(−7.170)	(−7.547)	(−4.816)
Roa	0.218	0.055	0.074	0.360**	0.308**	0.001
	(0.700)	(0.206)	(0.289)	(2.178)	(2.205)	(0.010)
Growth	0.023	0.014	0.003	−0.032**	−0.027**	−0.035***
	(0.865)	(0.611)	(0.112)	(−2.529)	(−2.487)	(−3.464)
Size	0.576***	0.477***	0.444***	0.542***	0.453***	0.389***
	(29.634)	(27.214)	(24.541)	(47.388)	(43.854)	(38.220)
Listage	0.016	0.006	−0.027	0.008	0.002	−0.009
	(0.735)	(0.342)	(−1.502)	(0.589)	(0.217)	(−0.809)
Ppeta	−0.730***	−0.574***	−0.491***	−0.539***	−0.415***	−0.342***
	(−6.399)	(−5.955)	(−5.101)	(−7.737)	(−6.971)	(−5.911)
Share1	0.021	−0.014	0.105	−0.003***	−−0.003***	−0.001
	(0.182)	(−0.140)	(1.041)	(−4.446)	(−4.354)	(−1.353)
Cfo	0.180	0.152	0.185	0.093	0.033	0.150
	(0.747)	(0.754)	(0.902)	(0.700)	(0.292)	(1.390)
Cash	−0.227*	−0.182*	−0.136	0.062	0.075	0.079
	(−1.793)	(−1.704)	(−1.279)	(0.837)	(1.191)	(1.331)
Esh	0.056	0.023	0.049	0.033	−0.002	0.038
	(0.636)	(0.321)	(0.701)	(0.667)	(−0.053)	(0.977)
imr2				0.109**	0.105**	0.055
				(2.162)	(2.458)	(1.303)
Indu	Yes	Yes	Yes	Yes	Yes	Yes
Year	Yes	Yes	Yes	Yes	Yes	Yes
Constant	−11.533***	−9.483***	−9.179***	−10.993***	−9.183***	−8.150***
	(−28.053)	(−25.603)	(−24.269)	(−42.720)	(−39.916)	(−36.508)
N	7081	7081	7081	20834	20834	20834
Adj-*R*^2^	0.276	0.262	0.250	0.237	0.222	0.211

T-values in parentheses, *p < 0.1, **p < 0.05, ***p < 0.01.

#### Heckman’s two-stage test

The Heckman two-stage regression is used for robustness testing to address possible omitted variables. In the first stage, the inverse Mills ratio (*imr2*) is calculated using the regression results estimated by the probit model. This is based on whether executives have experienced poverty (Famine) as the explained variable, and introducing the proportion of executives with experience of poverty in other companies in the industry as exogenous tool variables, plus all the control variables included in the main regression. In the second stage, the *imr2* calculated in the first stage is incorporated into the second stage regression model for fitting purposes. The specific regression results are presented in Columns (4–6) of Table 8. Even though the coefficient of *imr2* is significant in the second stage of analysis, the regression coefficient of Famine is still significant, indicating that the positive relationship between executive poverty experience and corporate innovation performance is robust after controlling for the omitted variable problem; supporting the conclusions of this study.

#### Variation substitution test

We selected four alternative indicators of innovation performance to avoid the singularity of innovation performance research indicators to increase the robustness of our findings. It takes an average of 2–3 years for general invention patents to be granted but less than 1 year for other patents because of the significant differences in the length of time from filing to grant. Consequently, the variable Gpatent1 is set to calculate the natural logarithm of the total number of invention patents granted within 3 years of the patent application; the variable Gpatent2 is calculated using the natural logarithm of the total number of utility and design patents granted within 1 year of application. Moreover, this study examines innovation performance but also allows for some lag between R&D and innovation output to better capture the impact of executives on R&D investment. Therefore, the alternative variable R&D1 is defined as the natural logarithm of a company’s R&D expenditure. The alternative variable R&D2 is defined as R&D expenditure divided by total assets of the company. The regression results, presented in Table 9, indicate that the regression coefficients of Famine and Gpatenth1, Gpatenth2, R&D1, and R&D2 are 0.058, 0.064, 0.175, and 0.001, respectively; all are significantly positive at the 1% level, indicating the robustness of previous research findings.

**TABLE 9:** Alternative measures of variables.

	(1)	(2)	(3)	(4)
	Gpatenth1	Gpatenth2	R&D1	R&D2
Famine	0.058***	0.064***	0.175***	0.001***
	(3.112)	(3.015)	(3.870)	(4.849)
Leve	−0.260***	−0.220***	−0.207	0.002***
	(−6.090)	(−4.231)	(−1.253)	(2.962)
Roa	0.042	0.012	−0.287	0.006***
	(0.353)	(0.078)	(−0.585)	(2.862)
Growth	−0.014	−0.039***	0.095***	0.000***
	(−1.412)	(−3.134)	(2.682)	(3.818)
Size	0.315***	0.449***	0.533***	−0.001***
	(32.054)	(39.951)	(18.723)	(−7.014)
Listage	0.028***	0.012	−0.327***	−0.001***
	(3.036)	(1.105)	(−10.357)	(−9.497)
Ppeta	−0.427***	−0.404***	−1.467***	−0.006***
	(−7.886)	(−6.239)	(−6.880)	(−9.631)
Share1	0.077	0.084	−0.486***	−0.002***
	(1.417)	(1.313)	(−2.961)	(−3.577)
Cfo	0.170*	0.042	2.030***	0.015***
	(1.666)	(0.331)	(4.669)	(10.126)
Cash	−0.096*	0.025	−1.100***	0.001
	(−1.663)	(0.380)	(−5.460)	(1.042)
Esh	0.016	0.016	0.235***	0.000
	(0.432)	(0.368)	(2.619)	(0.528)
Indu	Yes	Yes	Yes	Yes
Year	Yes	Yes	Yes	Yes
Constant	−6.233***	−9.486***	−10.319***	0.010***
	(−29.977)	(−40.278)	(−17.813)	(5.522)
N	20834	20834	20834	20834
Adj-*R*^2^	0.233	0.222	0.879	0.519

T-values in parentheses, *p < 0.1, **p < 0.05, ***p < 0.01.

## Discussion

Innovation plays an increasingly important role in maintaining enterprise competitiveness and improving performance; additionally, the impact of enterprise executives on innovation has also been recognized by scholars (Raffaelli et al., 2019). An increasing number of scholars have begun to pay attention to the relationship between executive characteristics and innovation performance and how executives with different background characteristics view the role of innovation risk in the innovation process (Chen and Nadkarni, 2017; Kiss et al., 2021). However, existing studies pay more attention to marital status (Roussanov and Savor, 2014), political relations (Hutton et al., 2014), professional background (Schoar and Zuo, 2017), senior executives at the later stage of their growth, and pay less attention to the impact of their early experiences on innovation activities.

Based on theoretical frameworks such as upper echelons theory and imprint theory, we studied whether the early life experience of senior executives in the Great Chinese Famine affected their company’s innovation performance. The results show that companies with executives who have famine experience produce better innovation results than those without. Adamkovič and Martončik (2017) showed that poverty affects economic decision-making via cognitive load, executive functions, and intuitive thinking styles. However, the effect of poverty experience on innovation behavior has not been further analyzed.

From the perspective of risk, innovation is a high-risk investment activity, and people who conduct innovation activities should have a certain risk preference. People affected by poverty are more reluctant to take risks, and prefer deterministic financial incentives (Haushofer and Fehr, 2014). However, a person who has experienced poverty needs to make more effort to become an enterprise executive. According to this study, people who have experienced poverty may have a greater change in their risk attitude in the process of becoming a senior executive and can more fully understand the relationship between risk and return.

In addition, in the case of two-rights separation, executives are motivated to damage the interests of shareholders for their own interests, in which earnings management is a common opportunistic behavior. In the R&D process, enterprises exhibit R&D manipulation behavior, which affects innovation performance. Yang et al. (2017) found that R&D regulation is positively correlated with tax benefits and government subsidiaries received by enterprises, whereas existing studies pay less attention to the impact of executive ethics on corporate R&D manipulation. Feng and Johansson (2018) find that CEOs who experienced the Great Chinese Famine in their youth were less likely to commit fraud and showed a higher level of ethics. Therefore, this study tests whether executives with poor experiences inhibit R&D manipulation. This conclusion shows that executives with high moral quality improve innovation performance by reducing R&D manipulation.

As for research on female executives, many studies prove that female executives are risk-averse and that the company’s decision-making is more conservative (Maxfield et al., 2010; Cumming et al., 2015; Chen et al., 2016). Therefore, female executives may reduce innovation expenditures and enterprise innovation performance. The results of this study shows that the innovation performance of female executives is better and argues that the reasons for the high innovation performance of enterprises should be analyzed from multiple perspectives including risk. The steady behavior style and higher moral quality of female executives are conducive to improving the success rate of enterprises’ R&D. In addition, by classifying patents, this study found that female executives have a higher impact on innovation types with lower risk. Therefore, the low-risk preferences of female executives may manifest more in different types of innovation activities.

Finally, compared with non-state-owned enterprises, there are great differences in management objectives, corporate governance models, and incentive assessment schemes of SOEs. Belloc (2014) believes that the innovation efficiency of SOEs is low because of the differences in property rights. This study finds that in SOEs, the role of senior executives’ human capital, which is also an important factor affecting the innovation performance of SOEs, has not been fully played.

## Conclusion

Personal experience affects the knowledge structure, cognitive model, and value orientation of executives, which then affects the strategic decision-making of enterprises. Poverty experiences are imprinted on executives’ sub consciousness, which largely affect their risk appetite and behavioral decision-making. Existing research on how poverty experience affects executives’ decision-making when faced with risky innovation activities lacks in-depth analysis and empirical research. Therefore, we propose a comprehensive research framework to analyze the influence mechanism of executives’ early life poverty experiences on innovation performance.

Based on the data of Chinese A-share listed companies from 2012 to 2020, the empirical results show that executives who have experienced the Great Chinese Famine significantly improve corporate innovation performance. This study discusses the boundary of poverty experience affecting enterprise innovation from the perspective of gender, the nature of property rights, and market competition. Further, study on the influence mechanism revealed that executives who experience poverty during their early life have a stronger sense of morality and responsibility, which inhibits corporate R&D manipulation and can therefore positively affect the organization’s innovation performance. This study enriches the research on the factors influencing enterprise innovation performance and combines the theories of psychology and sociology to provide empirical evidence for improving enterprise innovation performance.

### Theoretical implications

Theoretically, this study finds that executive poverty experience plays a vital role in corporate innovation performance from the perspective of early life executive experience, and verifies the boundary and mechanism of its influence, which provides exceptional insight into the factors affecting corporate innovation performance. It also provides empirical support for improving corporate innovation performance by combining psychological and sociological theories.

This study provides an in-depth analysis of the impact of executive background experiences on corporate innovation, supporting and enriching the existing literature. Existing studies related to poverty experience focus more on the effects of micro-firm investment and financing, social responsibility, and financial asset allocation (Malmendier et al., 2011; Bernile et al., 2017; O’Sullivan et al., 2021). Using previous research, this study investigates the impact of executives’ childhood experiences of the Great Chinese Famine on corporate innovation using data from Chinese A-share listed companies. It analyzes the overall innovation level of enterprises by focusing on the variables of enterprise innovation as well as explores group regression based on the innovation of patent categories, explores the differences in the impact of poverty experiences on different innovation types of enterprises, and improves relevant research conclusions.

Further, we contribute to relevant research by examining and discussing the effects of poverty in terms of heterogeneity regarding gender, property rights, and market competition on firm innovation. We find that female executives from poor backgrounds abandon traditional female “conservative” risk preferences (Faccio et al., 2016). Moreover, female executives from poor backgrounds have greater risk-taking abilities and courage due to their experience in the business world. An analysis of the nature of property rights finds that operating pressures specific to SOEs have a negative impact on corporate innovation and that executive poverty experience plays a more significant role in non-state-owned enterprises. The competitive market environment faced by firms can also moderate the role of executive poverty experience to varying degrees.

Finally, this study clarifies the mechanism behind the impact of executive poverty experience on corporate innovation from the perspective of R&D manipulation through a mediating effects model. The literature on the effect of executive background on innovation performance is more likely to develop mechanistic studies from the perspective of corporate risk-taking than from an individual perspective. A controversial issue is the impact of executive poverty on corporate risk-taking (Stephens et al., 2014; Bernile et al., 2017; Hu et al., 2020). Thus, this study builds on the fact that executive poverty experience can increase their sense of morality and responsibility and empirically finds that executive poverty experience can inhibit R&D manipulation behavior. Further, it plays a partial mediating role in the effect of executives’ early life poverty experiences on corporate innovation, expanding research relevant to the subject, and supporting Feng and Johansson’s conclusions.

### Practical implications

In this new era, innovation has emerged as a key component in accelerating the economic development of nations. In addition, innovation facilitates the “deepening of structural reform on the supply side” and accelerates the construction of a truly innovative nation. The practical implications of this study are as follows.

First, executives’ early life experiences can profoundly influence their behavioral decisions. Therefore, it is important to pay attention to candidates’ background experiences when selecting and hiring executives, as well as to fully understand the role of personality traits in organizational behavior. According to strategic planning, an enterprise should increase the selection of senior executives with a “matching degree” to minimize the trial-and-error cost of the enterprise. In the recruitment process, executive applicants with impoverished backgrounds should follow the principles of “competency matching,” regardless of the origin, and pay attention to their ability and moral quality. In addition, the early life experiences of executives may be influenced by individual, corporate, and market characteristics, and it is necessary to examine a variety of factors rather than relying solely on one when analyzing the behavior of executives.

Second, the government should actively improve the corporate governance system of SOEs, optimize the employment system, and fully stimulate the positive role of senior executives in innovation. This study finds that in SOEs, the positive impact of executives with poverty experience on innovation is weakened. This may be related to corporate governance factors such as the assessment system of SOEs. The government should actively formulate reform policies and improve the corporate governance structure to improve the efficiency and effect of innovation in SOEs. Only by promoting a market-oriented personnel selection mechanism and issuing supporting compensation incentive policies can SOEs continuously stimulate their innovation vitality.

Finally, this study finds that there is R&D manipulation in the process of enterprise innovation, and that enterprise innovation performance is affected by the moral level of senior executives. Innovation activities of enterprises are important references for capital market valuation. R&D manipulation is detrimental to the stable development of enterprises, reduces the efficiency of the capital markets, and damages investors’ interests. The government should punish and regulate enterprises’ behaviors that harm public interest through R&D manipulation. For enterprises with high executive ethics and less R&D manipulation, the government should provide policy support, including financial subsidies, government investment, government procurement, and government approval.

### Limitations and future research

This study has some limitations and provides additional opportunities for future research. First, due to data limitations, our research results may be limited to Chinese listed companies. The listing conditions for Chinese enterprises are quite strict and need to be reviewed by the Securities Supervision Commission (Tian, 2011; Li Y. et al., 2018; Xiaoyan and Lianghua, 2021). A person needs to have comprehensive qualities in all aspects to become an executive of a listed company in China. The impact of early poverty experience on executives of listed companies may limit the universality of our research results. Future research can focus on the performance of people with poverty experience in unlisted companies, and the research object can also be a broader group of executives.

Additionally, it is difficult to obtain accurate data collection and analysis to assess the level of executive poverty experience. Although the Great Chinese Famine of 1959 to 1961 can generally be approximated, a more detailed and in-depth analysis of the research problems may be helpful if there is a better alternative measurement method. Whether executives were born in distressed area, disadvantaged families, poor countries, etc. can measure whether they have early poverty experience.

Third, there are many types of innovation activities. This paper focuses on invention patents, utility models patents and designs patents. Innovation activities can also be divided into independent innovation and open innovation. With the increasing complexity and uncertainty of innovation, open innovation has gradually become the mainstream mode of innovation. Open innovation has the characteristics of risk sharing and achievement sharing. This model emphasizes the interdependence and cooperation of multi organizations in innovation activities (Saura et al., 2022). What impact the early experiences of executives will have on open innovation is still a question worthy of in-depth study.

Finally, this study examines the influence mechanism of R&D manipulation; however, we believe that there may be other ways to influence senior executives’ early life poverty experiences, which need to be explored in the future. And, this study found that factors such as the background characteristics of executives, business characteristics, and market characteristics have specific differences in the effects of innovative patents. Thus, what may cause these differences and how to promote highly innovative patent development need to be further examined in the future.

## Data availability statement

The raw data supporting the conclusions of this article will be made available by the authors, without undue reservation, to any qualified researcher.

## Author contributions

All authors listed have made a substantial, direct, and intellectual contribution to the work, and approved the final manuscript. XJ: research ideas, concept and design, obtaining funding, statistical analysis, interpretation of data, and study supervision. TW: data curation, concept and design, statistical analysis, and interpretation of data. CC: research ideas, concept and design, data curation, and writing up.
